# Predicting Nonlinear and Anisotropic Mechanics of Metal Rubber Using a Combination of Constitutive Modeling, Machine Learning, and Finite Element Analysis

**DOI:** 10.3390/ma14185200

**Published:** 2021-09-10

**Authors:** Yalei Zhao, Hui Yan, Yiming Wang, Tianyi Jiang, Hongyuan Jiang

**Affiliations:** School of Mechatronics Engineering, Harbin Institute of Technology (HIT), Harbin 150001, China; zhaoyl@hit.edu.cn (Y.Z.); 20s108328@stu.hit.edu.cn (Y.W.); jty_hit@hit.edu.cn (T.J.)

**Keywords:** nonlinearity, anisotropy, metal rubber, ANN, mechanics prediction

## Abstract

Metal rubber (MR) is an entangled fibrous functional material, and its mechanical properties are crucial for its applications; however, numerical constitutive models of MR for prediction and calculation are currently undeveloped. In this work, we provide a numerical constitutive model to express the mechanics of MR materials and develop an efficient finite elements method (FEM) to calculate the performance of MR components. We analyze the nonlinearity and anisotropy characteristics of MR during the deformation process. The elasticity matrix is adopted to express the nonlinearity and anisotropy of MR. An artificial neural network (ANN) model is built, trained, and tested to output the current elastic moduli for the elasticity matrix. Then, we combine the constitutive ANN model with the finite element method simulation to calculate the mechanics of the MR component. Finally, we perform a series of static and shock experiments and finite element simulations of an MR isolator. The results demonstrate the feasibility and accuracy of the numerical constitutive MR model. This work provides an efficient and convenient method for the design and analysis of MR components.

## 1. Introduction

Entangled fibrous materials are emerging materials [1,2,3,4,5,6]. The form of the material, such as fiber, wire, or helix spring, is reconstructed through entanglement with itself to achieve new physical properties without changing the chemical composition. These materials are called architectured or architected because their mechanical properties strongly depend on the geometry of their internal structure after scaling [7]. For example, metal wools are composed of free curved metal fibers (25 μm) by rolling up and pressing in the mold, which can be used as electrical vias in soft devices or soft sensors [8]. The metal mesh is made of knitted wire mesh and is usually shaped by compression after folding up. As a commercial functional material, the metal mesh is widely used in dampers, filters, and seals [9,10,11,12]. Unlike the wire inside metal mesh, which is entangled by folding and squeezing, metal rubber (MR) consists of a mass of tiny metal helix springs entangled with each other via embedding and interlacing during the forming process. This entangled pattern of the tiny metal springs results in improved elasticity and mechanical properties; therefore, the MR components show excellent performance in engineering applications [13,14,15,16,17,18].

Metal rubber materials are designable; the mechanics change with the relative density (the ratio of metal rubber’s true density to the metal wire’s density, and it is determined by measuring the mass of MR whose volume is constant), maximum loading strain, and diameter of the metal wire. MR’s mechanics is the essential factor in the design of MR components. However, due to the arrangement pattern of the inner micro springs, the mechanics of MR shows typical nonlinearity and anisotropy, which makes it difficult to calculate and predict the performance of MR components, such as the static stiffness and shock response of the MR isolator. The mechanics of MR materials has been studied by many researchers [19,20,21,22,23]. Hong et al. [24] and Wang et al. [25] reported the effects of relative density, displacement, and wire diameter on the stiffness and loss factor of MR. Yang et al. [26] and Ren et al. [27] studied the effects of relative density and wire diameter on the damping capability of MR in the no-molding direction. On the basis of virtual manufacturing technology, Ren et al. [28] studied the mechanical properties of MR and reported that the wire diameter was one of the most important factors. Hu et al. [29] presented that the tangent modulus and loss factor of multiple wire MR changed as the weight percentage ratio varies.

Generally, we identify satisfactory material parameters through multiple tests during the design of MR components. The finite element method (FEM) is an economical and efficient method used to analyze the MR components, especially under the circumstances of complex structural analysis and as a substitute for expensive experiments. The corresponding numerical constitutive model, which is used to describe the nonlinearity and anisotropy of MR materials with different parameter combinations, is indispensable for the accurate performance calculation and prediction of MR components.

Zhu et al. [30] analyzed three types of contact status of micro helix springs under the assumption that the spatial distribution of the microelements inside the MR is even and periodic. On that basis, they built a mathematical model to describe the behavior of the MR. Ma et al. [31] introduced MR into the tensegrity structure to improve energy absorption and reduce the stiffness, and they presented a mathematical model of tensegrity based on the nonlinearity mechanics of MR. The pyramid model was used to describe the principle of energy dissipation of MR. Before calculating the horizontal and vertical friction pair, it is necessary to experimentally identify the coefficient of the MR and the angle of the pyramid contact pair [32]. The flexural-cantilever model was used to simulate the contact states between the micro springs as a series of cantilevers contacted at the free end. The MR was considered as a structural damping system consisting of parallel trussing and series-connected micro springs [33]. For the flexural cantilever model, the major coefficients are the number of micro-units, contact points, and the length of the cantilever. These models express the mechanics of MR by describing the interaction between the entangled micro springs. However, MR’s internal structure needs a large number of elements and nodes to be accurately expressed in the FEM simulation, and the complex interaction between micro springs may also cause problems of convergence. These models, which need to consume plenty of memory and computational time, are not efficient for FEM.

Machine learning has been used to build constitutive models of materials that exhibit nonlinear behavior [34]. Artificial neural networks (ANNs) have many advantages, such as efficiency and ease of implementation. ANNs are convenient for accurate prediction and numerical simulation because they ignore all the processes and theories; thus, they have been widely used to study material properties [35,36,37,38,39,40,41,42,43,44,45,46,47,48]. The combination of machine learning and FEM has been used to solve complex engineering problems [49,50,51,52]. Hashash et al. [53] addressed numerical implementation issues related to ANN constitutive models in FEM analysis. They derived a consistent material stiffness matrix for the ANN constitutive model that leads to efficient convergence of the FE Newton iterations. To describe the nonlinear elastic modulus pattern of the L-bending springback process, Jamli et al. [54] developed an ANN-based material constitutive model by combining ANN pattern recognition and FEM code. However, ANNs have rarely been used to describe the feature of MR materials.

In this work, for accurate performance prediction and calculation of MR components, we developed a numerical constitutive model of an MR material based on machine learning. First, we analyzed the nonlinearity and anisotropy of the MR material and developed an elasticity matrix to express these features. The major variables of the elasticity matrix were the current elastic moduli. Second, we built an ANN model to update the current elastic modulus and combine it with FEM to conduct numerical calculations. Finally, a series of experiments and FEM simulations were performed under the same conditions. The simulation and experimental results showed good agreement, indicating the feasibility and accuracy of the proposed MR model. This work provides an efficient and convenient method for the design of MR components.

## 2. Materials and Methods

### 2.1. Mechanical Properties of MR

The MR material is made of a mass of micro helical springs, and its basic element is metal wire. The mechanics of MR depends on the properties of metal wire and micro helical springs. Consequently, there are two types of parameters of MR: parameters of wire materials and parameters of structure. The property of wire materials certainly influences the mechanics of MR. In this study, we focus on the MR made of 321 stainless steel (China Grand 1Cr18Ni9Ti, Gaona Aero Material Co., Ltd., Beijing, China). The structure parameters of MR include the diameter of the micro helical spring, the diameter of the wire, and the relative density. For the MR damper, the relationship between diameters of the micro helical spring and metal wire is generally set as 10 times.

MR is an elastic material, and its common work pattern is compressed under the loading stage and bounced back during the unloading stage. During these processes, the mechanical properties of MR show typical anisotropy and nonlinearity.

#### 2.1.1. Anisotropy of MR

**Figure 1:**
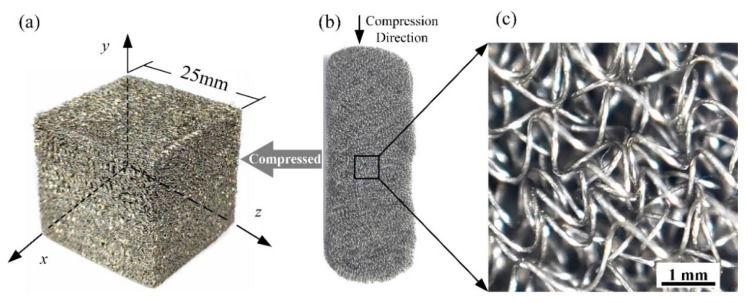
The forming process of MR: (**a**) MR cube with 25-mm sides, (**b**) workblank of the MR cube, (**c**) micro springs of the workblank.

The anisotropy of MR originates from the forming process. Figure 1a shows an MR cube with 25-mm sides, and Figure 1b shows its workblank. The workblank is formed in a rigid mold by compression. Figure 1c shows a partial enlargement of the workblank, illustrating that the distribution of micro springs is random.

During the compression process, the micro springs, as shown in Figure 1c, will overlap with each other, and the movements of micro springs in the direction perpendicular to the compressing direction are constrained by the mold. As shown in Figure 1, for the MR cube, the *y*–direction is the compression direction, and the *x* and *z*–directions are constrained directions. The movement of micro springs in the *y*–direction leads to the corresponding different arrangement pattern of micro springs in the *x*–*y*, *y*–*z*, and *x*–*z* planes.

**Figure 2:**
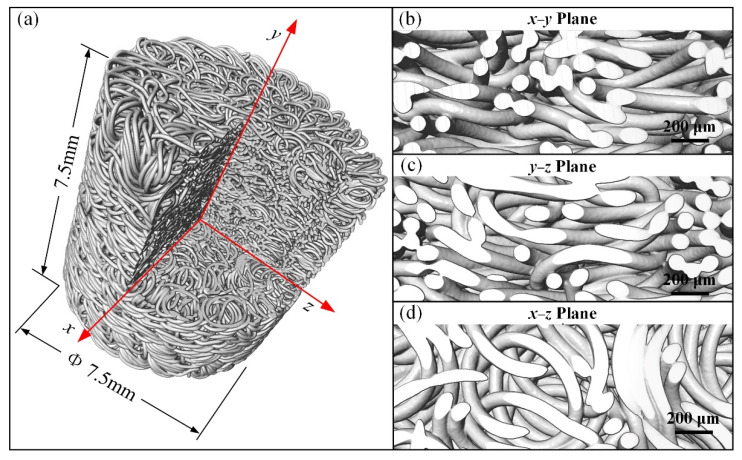
3D X-ray CT microscopy images of MR cylinder: (**a**) reconstructed MR cylinder image with a corner cut out, (**b**) cross-section enlargement image in the *x*–*y* plane, (**c**) cross-section enlargement image in the *y*–*z* plane, (**d**) cross-section enlargement image in *x*–*z* plane.

The 3D microscopy technology helps us to observe the inner structures of the MR. Figure 2 shows the 3D X-ray CT microscope images (captured by a ZEISS Xradia 520 Versa, Carl Zeiss X-ray Microscopy Inc., Dublin, CA, USA) of an MR cylinder with a height and diameter of 7.5 mm. Figure 2a presents the image of the reconstructed MR cylinder with a corner cut out. The cross-sectional images of the MR cylinder reveal the different entanglement patterns of the micro springs. Figure 2b–d is the partial enlargement images of the cross-section of the MR cylinder in the *x*–*y*, *y*–*z*, and *x*–*z* planes, respectively. The arrangement of micro springs in the *x*–*y* plane is shown in Figure 2b, and its main entangled pattern of micro springs are overlapped with each other in the axial direction. The entanglement pattern in the *x*–*y* and *y*–*z* planes are similar. The arrangement of micro springs in the *x*–*z* plane is shown in Figure 2d, and its main entangled pattern of micro springs is embedded in the radial direction.

**Figure 3:**
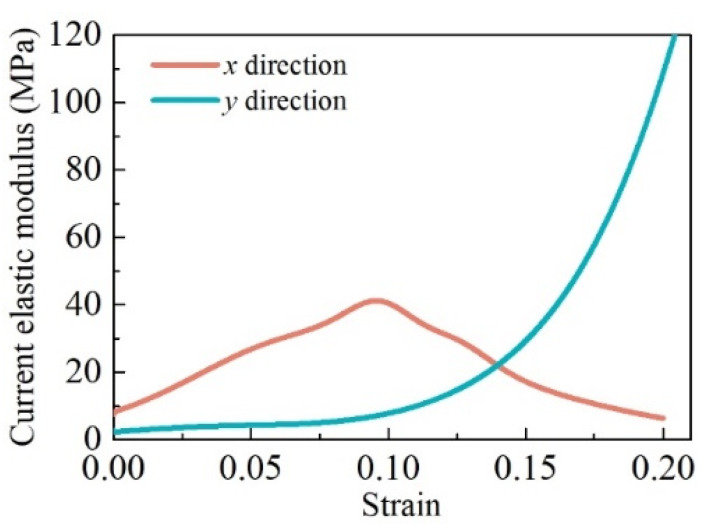
Difference in current elastic modulus in *x* and *y*–directions.

Because of the different distribution patterns of micro springs, the mechanical property of MR in the *x*–direction was the same as that in the *z*–direction but different from that in the *y*–direction. Figure 3 shows the uniaxial experimental results of the MR cube in the *x* and *y*–directions at the loading stage. For the experiments, the relative density of the MR cube was 0.28, and the metal wire diameter was 0.1 mm. There is a distinct difference between the trend of the current elastic modulus in the *x* and *y*–directions. The current elastic modulus in the *y*–direction increases with strain. In the beginning, the elastic modulus in the *x*–direction was larger than that in the *y*–direction; however, this trend inverted with increasing strain. Therefore, MR is a classical transversely isotropic material, and the plane perpendicular to the shaping direction, as shown in Figure 1, is the isotropic plane. Ma et al. [10] observed and quantified the transverse isotropy of the wire orientation in metal rubber by using the visualization skeleton model, and they had the same conclusion.

According to Ma et al. [55], the cross-sections of MR do not affect mechanical properties, and the main size effect of MR is the boundary layer effect, which is reflected in the height of the MR components. However, the boundary layer effect is negligible when MR components are higher than 10 mm. In this work, we do not consider the size effect of MR.

#### 2.1.2. Nonlinearity of MR

**Figure 4:**
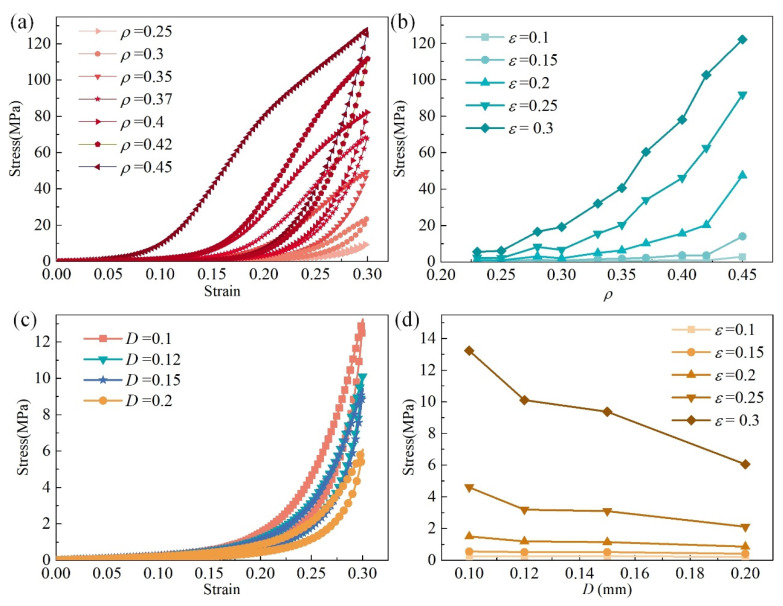
Stress–strain properties of MR: (**a**) stress–strain property for different relative densities, (**b**) stress nonlinearity between different relative densities, (**c**) stress–strain property for different wire diameters, (**d**) stress nonlinearity between different wire diameters.

**Figure 5:**
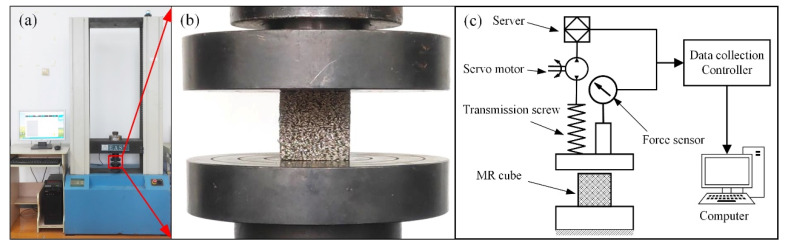
Uniaxial compression experiments of MR cube: (**a**) universal testing machine; (**b**) MR cube; (**c**) schematic of the compression experiment.

MR is a typical nonlinear material. Figure 4 presents the uniaxial compression experiment results of MR cubes with different parameters in the *y*–direction. The uniaxial compression experiments were carried out by WDW-100 electronic universal testing machine (Jinan East Testing Machine Co., Ltd., Jinan, China), as shown in Figure 5a. Figure 5b shows the detail of the MR cube in the uniaxial compression experiment. The schematic of the experiment is shown in Figure 5c.

The nonlinearity characteristic of MR can be observed from the strain–stress curve, as shown in Figure 4a. The stress of MR mainly consists of two parts; one part comes from the elastic-plastic deformation of the micro springs, and another part from the friction due to the relative motion between micro springs. The arrangement of inner micro springs is irregular, and the contact points and contact status are difficult to count accurately. We intend to solve this problem with a numerical constitutive material model.

The mechanics of MR shows multi-nonlinearity. On the one hand, the contact points and micro springs contact status inside of the MR will change with increasing deformation. On the other hand, the nonlinearity of stress is directly affected by the parameters of the MR, such as the metal wire diameter, the relative density, and the material of the metal wire. Figure 4a presents strain–stress curves of MR cubes with different relative densities, *ρ* = 0.25, 0.3, 0.35, 0.37, 0.4, 0.42, and 0.45, where the metal wire diameters are the same, *D* = 0.12 mm. The curves show that the stress and the area of the loop increase with increasing relative density. For the same strain, the stress increases with increasing relative density, and the trend shows nonlinearity, as shown in Figure 4b.

The stress–strain curves shown in Figure 4c were obtained from MR cubes with the same relative density, *ρ* = 0.25, but different metal wire diameters, *D* = 0.1, 0.12, 0.15, and 0.2 mm. The stress mainly decreases with increasing metal wire diameter. Figure 4d shows the trend of the stress changing with metal wire diameters for a strain of 0.1, 0.15, 0.2, 0.25, and 0.3.

### 2.2. Current Elastic Modulus of MR

According to previous research, we know that different sizes of MR material show the classical strain–stress relationship. The strain–stress relationship can be expressed by the generalized Hooke’s law, as shown in Equation (1). Therefore, we can use the elastic modulus of each strain point, the current elastic modulus, to describe the nonlinearity property of MR, as shown in Equation (2).

(1) $$E=\frac{\sigma }{\varepsilon },$$

(2) $$E_{C}=\frac{\Delta \sigma }{\Delta \varepsilon },$$

where *E* is the elastic modulus, *σ* is the stress, *ε* is the strain, *E*_C_ is the current elastic modulus, Δ*σ* is the stress increment, and Δ*ε* is the strain increment.

**Figure 6:**
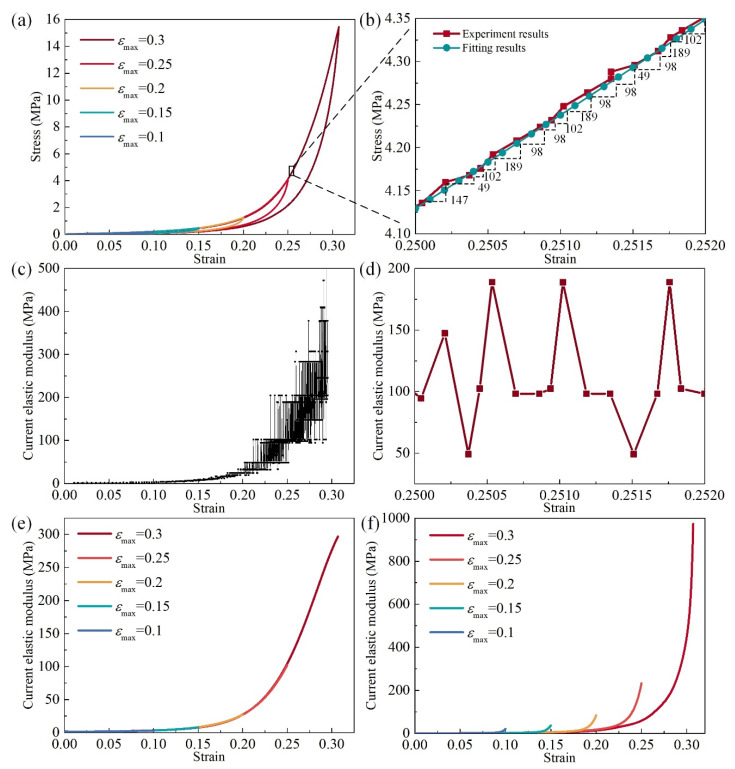
Current elastic modulus of MR: (**a**) stress–strain property of different maximum loading strains, (**b**) enlargement of stress–strain curve (**a**) and slope of each strain point, (**c**) elastic modulus of the experimental stress–strain results, (**d**) enlargement of (**c**), (**e**) smoothed elastic modulus of loading stage with different maximum strain, (**f**) smoothed elastic modulus of the unloading stage with different maximum strain.

Figure 6a presents strain–stress curves of an MR cube with the relative density fixed at 0.26 for a max strain of 0.1, 0.15, 0.2, 0.25, and 0.3. As observed in Figure 6b, the slope of the loading process is the current elastic modulus. Owing to the sensor error during the data collection, the original stress–strain data was not smooth in detail. Although the errors of stress–strain data are not significant, the errors of slopes are magnified in each strain incremental step, as shown in Figure 6c,d. In order to eliminate the errors of slopes, the strain–stress curve needed to be smoothed. The original strain–stress data are fitted by using a simple ANN, which has two layers (4 neurons for the hidden layer and 1 for the output layer). The input of the ANN, which is trained by the original strain-stress data, are regular strains, and the outputs are regular and smooth strain–stress data, as shown in Figure 6b. The excellent fitting function of ANN makes sure that the true nonlinearity of the strain–stress is preserved. In this work, the machine learning activities are performed in the python environment. Figure 6e,f shows the curves of the current elastic modulus of the loading and unloading stage under different maximum strains.

When MR is used as a damping material, it consumes the vibration energy because of the typical hysteresis characteristics of the loading–unloading loop. The nonlinear mechanics of MR exists both at the loading and unloading process. For the loading stage, the strain–stress curves of the MR under different maximum strains are consistent; however, for the unloading stage, the nonlinear feature changes with the maximum strains.

### 2.3. Principle of the Constitutive Model

According to Hooke’s law, the relationship between strain and stress can be expressed by the elasticity matrix, as shown in Equation (3). Equation (4) is the inverse elasticity matrix of isotropic materials, and for orthotropic materials, it can be expressed by Equation (5).

(3) $$\left\{\begin{array}{c} \begin{array}{c} \begin{array}{c} \begin{array}{c} \sigma_{x}\\ \sigma_{y} \end{array}\\ \sigma_{z}\\ \tau_{xy} \end{array}\\ \tau_{yz} \end{array}\\ \tau_{zx} \end{array}\right\}=J\left\{\begin{array}{c} \begin{array}{c} \begin{array}{c} \begin{array}{c} \varepsilon_{x}\\ \varepsilon_{y} \end{array}\\ \varepsilon_{z}\\ \varepsilon_{xy} \end{array}\\ \varepsilon_{yz} \end{array}\\ \varepsilon_{zx} \end{array}\right\},$$

(4) $$J^{-1}=\left\{\begin{array}{cccccc} \frac{1}{E} & -\frac{\nu }{E} & -\frac{\nu }{E} & 0 & 0 & 0\\ -\frac{\nu }{E} & \frac{1}{E} & -\frac{\nu }{E} & 0 & 0 & 0\\ -\frac{\nu }{E} & -\frac{\nu }{E} & \frac{1}{E} & 0 & 0 & 0\\ 0 & 0 & 0 & \frac{1}{G} & 0 & 0\\ 0 & 0 & 0 & 0 & \frac{1}{G} & 0\\ 0 & 0 & 0 & 0 & 0 & \frac{1}{G} \end{array}\right\},$$

(5) $$J^{-1}=\left\{\begin{array}{cccccc} \frac{1}{E_{x}} & -\frac{\nu_{yx}}{E_{y}} & -\frac{\nu_{zx}}{E_{z}} & 0 & 0 & 0\\ -\frac{\nu_{xy}}{E_{x}} & \frac{1}{E_{y}} & -\frac{\nu_{zy}}{E_{z}} & 0 & 0 & 0\\ -\frac{\nu_{xz}}{E_{x}} & -\frac{\nu_{yz}}{E_{y}} & \frac{1}{E_{z}} & 0 & 0 & 0\\ 0 & 0 & 0 & \frac{1}{G_{xy}} & 0 & 0\\ 0 & 0 & 0 & 0 & \frac{1}{G_{yz}} & 0\\ 0 & 0 & 0 & 0 & 0 & \frac{1}{G_{zx}} \end{array}\right\},$$

where, *J* is elasticity matrix, *ν* is Poisson’s ratio, *G* is the shear modulus, *E_x_*, *E_y_*, and *E_z_* are the elastic moduli in the *x*, *y*, and *z*–directions, respectively, *ν_xy_*, *ν_xz_*, *ν_yx_*, *ν_yz_*, *ν_zy_*, and *ν_zx_* are Poisson’s ratio for different deformation states, and *G_xy_*, *G_yz_*, and *G_zx_* are shear moduli for *x*–*y*, *y*–*z*, and *z*–*x* planes, respectively.

Because MR is a transversely isotropic material, Equation (5) meets the conditions *E_x_* = *E_z_*, *ν_xy_* = *ν_zy_*, *ν_xz_* = *ν_zx_*, *ν_yx_* = *ν_yz_*, and *G_xy_* = *G_yz_*.

(6) $$G_{zx}=\frac{E_{x}}{2\left(1+\nu_{zx}\right)},$$

(7) $$G_{xy}=\frac{E_{y}}{2\left(1+\nu_{xy}\right)}.$$

If we identify the value of *E_x_*, *E_y_*, *ν_xy_*, *ν_yx_*, and *ν_zx_* for each relative strain point precisely, the mechanical properties of the MR material can be expressed accurately. Using this method, the stress of the MR for certain deformations can be determined using Equations (8) and (9).

(8) $$\left\{\begin{array}{c} \begin{array}{c} \begin{array}{c} \begin{array}{c} \sigma_{x}\\ \sigma_{y} \end{array}\\ \sigma_{z}\\ \tau_{xy} \end{array}\\ \tau_{yz} \end{array}\\ \tau_{zx} \end{array}\right\}=\frac{1}{\Delta }\left\{\begin{array}{cccccc} C_{11}E_{x} & C_{12}E_{x} & C_{13}E_{x} & & & \\ C_{21}E_{y} & C_{22}E_{y} & C_{21}E_{y} & & & \\ C_{13}E_{y} & C_{12}E_{x} & C_{11}E_{x} & & & \\ & & & \Delta G_{xy} & & \\ & & & & \Delta G_{yz} & \\ & & & & & \Delta G_{zx} \end{array}\right\}\left\{\begin{array}{c} \begin{array}{c} \begin{array}{c} \begin{array}{c} \varepsilon_{x}\\ \varepsilon_{y} \end{array}\\ \varepsilon_{z}\\ \varepsilon_{xy} \end{array}\\ \varepsilon_{yz} \end{array}\\ \varepsilon_{zx} \end{array}\right\},$$

(9) $$\left\{ \begin{array}{l} \Delta =1-2\nu_{xy}\nu_{yx}-\nu_{xz}{}^{2}-2\nu_{xy}\nu_{yx}\nu_{xz}\\ C_{11}=1-\nu_{xy}\nu_{yx}\\ C_{12}=\nu_{yx}+\nu_{yx}\nu_{xz}\\ C_{13}=\nu_{xz}+\nu_{xy}\nu_{yx}\\ C_{21}=\nu_{xy}+\nu_{xy}\nu_{xz}\\ C_{22}=1-\nu_{xz}{}^{2} \end{array}\right..$$

According to the experimental results of Wang et al. [31], the effective Poisson’s ratio *ν_yx_* was approximately zero. Zhang et al. [19] reported that the tangent Poisson’s ratio of MR was approximately 0.01 at small deformations and could reach 0.1 at large deformations. Wang [56] measured MR’s Poisson’s ratio in the *x*, *y*, and *z*–directions, and Wang’s work had a similar conclusion to Zhang et al. [19] and Wang et al. [31]. Therefore, in this work, we assume Poisson’s ratio is constant and *ν_xy_* = *ν_zy_* = 0.1 and *ν_xz_* = *ν_zx_* = *ν_yx_* = *ν_yz_* = 0.01.

### 2.4. Machine Learning Methodology

We intend to describe the nonlinear and anisotropic mechanics of MR components by capturing the current elastic modulus precisely for each strain point. Driven by this assumption, an ANN model is used to predict the current elastic modulus for MR with certain parameters.

For MR, building an ANN constitutive model consists of five major parts: problem representation, selecting the structure of the network, choosing a learning algorithm, using the database to train the ANN, and validating the performance of the trained ANN.

Considering the mechanical features of MR, we used the backpropagation (BP) neural network to build the ANN model. We adopted six major mechanical factors of MR as the inputs of the ANN model.

**Figure 7:**
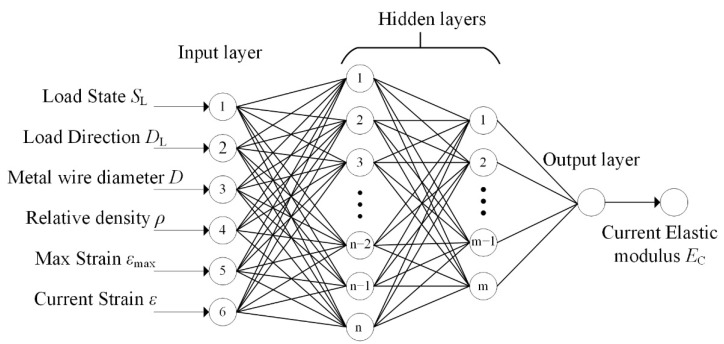
Architecture of the ANN model.

First, a complete hysteresis curve (mechanical property) of MR consists of the loading stage and unloading stage, and the unloading curve depends on the maximal loading strain. Second, MR is transversely isotropic, and its mechanical property in the *y*–direction is significantly different from that in the *x* or *z*–directions. Last, the mechanics of MR changes with wire diameter and relative density. Therefore, we adopted six major mechanical factors of MR as the inputs of the ANN model. The first input is the loading state *S*_L_; the loading stage is marked as 1, and the unloading stage is marked as −1. The second input is the loading direction *D*_L_; the *y*–direction is marked as 1, and the *x* and *z*–directions are marked as −1. The rest of the inputs include the metal wire diameter *D*, relative density *ρ*, maximum strain *ε*_max_, and current strain *ε*. The output of the ANN is the current elastic modulus *E*_C_. Figure 7 shows the structure of the ANN model.

The activation functions adopted for the hidden layers and output layer were tan-sigmoid and linear, respectively. The Levenberg–Marquardt algorithm was applied to train the ANN. The mean squared error (MSE) and coefficient of determination (*R*^2^), as described in Equations (10) and (11), were used to evaluate the training and predicting of the developed ANN model. MSE indicates the discrepancy between the experimental and calculated values. The lower the MSE, the more accurate the prediction. *R*^2^ measures the fitness of the model to the experimental data.

(10) $$\mathrm{MSE}\;=\;\sum_{1}^{n}\frac{\left(y_{i}-{\hat{y}}_{i}\right)}{n},$$

(11) $$R^{2}\;=\;1\;-\frac{\sum_{1}^{n}\left(y_{i}-{\hat{y}}_{i}\right)}{\sum_{1}^{n}\left(y_{i}-{\overset{-}{y}}_{i}\right)}.$$

## 3. Results and Discussion

### 3.1. ANN Model Training Results

First, we collected the strain–stress dataset from uniaxial compression mechanical tests of MR samples, which were cubes with 25-mm sides, as shown in Figure 1a. In this work, the metal wire material of the MR is 321 stainless steel, and the diameter of micro springs is 10 times that of the wire. The data of the current elastic modulus was obtained from the strain–stress data that was smoothed. In total, 44 groups of uniaxial compression experiments were carried out to collect the dataset, and the experiment equipment and method are shown in Figure 2. The details of the specimens are presented in Table 1. The 44 specimens included 4 diameters, which were the most commonly used to manufacture MR components, and the specimens of each diameter contained 11 relative densities, which covered the range commonly used in the MR components design. A total of 40 groups data (91%) were labeled as training data, and 4 groups data (9%) were labeled as testing data.

In order to configure the optimum ANN model and avoid overfitting, eight combination trials with different architectures were evaluated. The details of each trial, training for 2000 iterations, along with the results of MSE and *R*^2^ are shown in Table 2. MSE represents the accuracy of prediction, and *R*^2^ indicates the fitness of the model to the experimental data. According to Table 2, increasing neurons can get better training MSE and *R*^2^, but it may lead to increasing testing MSE and decreasing testing *R*^2^, which are the signs of overfitting. We avoided overfitting by controlling the number of neurons. The best model performance in terms of least testing MSE = 292 and the highest *R*^2^ = 0.9930 was obtained. The neural number of the first and second hidden layers are n = 24 and m = 12, respectively.

**Figure 8:**
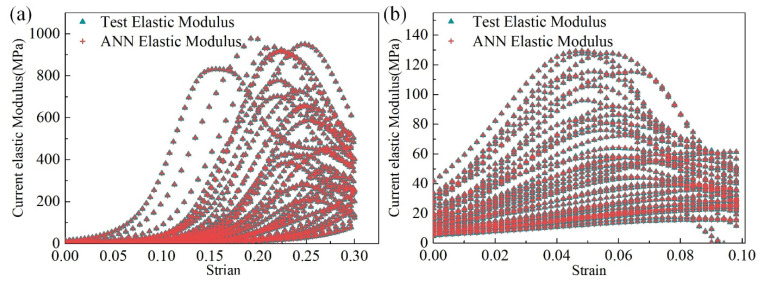
Comparison between training data and ANN model output: (**a**) training results of the loading stage in the y–direction and (**b**) training results of the loading stage in the x–direction.

Figure 8a compares the elastic modulus in the loading stage and *y*–direction obtained from the trained ANN model and the original training dataset. Similarly, Figure 8b presents the elastic modulus outputted by the trained ANN model in the loading stage and *x*–direction (or *z*). The output of the trained ANN model was consistent with the original training dataset.

### 3.2. Validity of ANN Model

**Figure 9:**
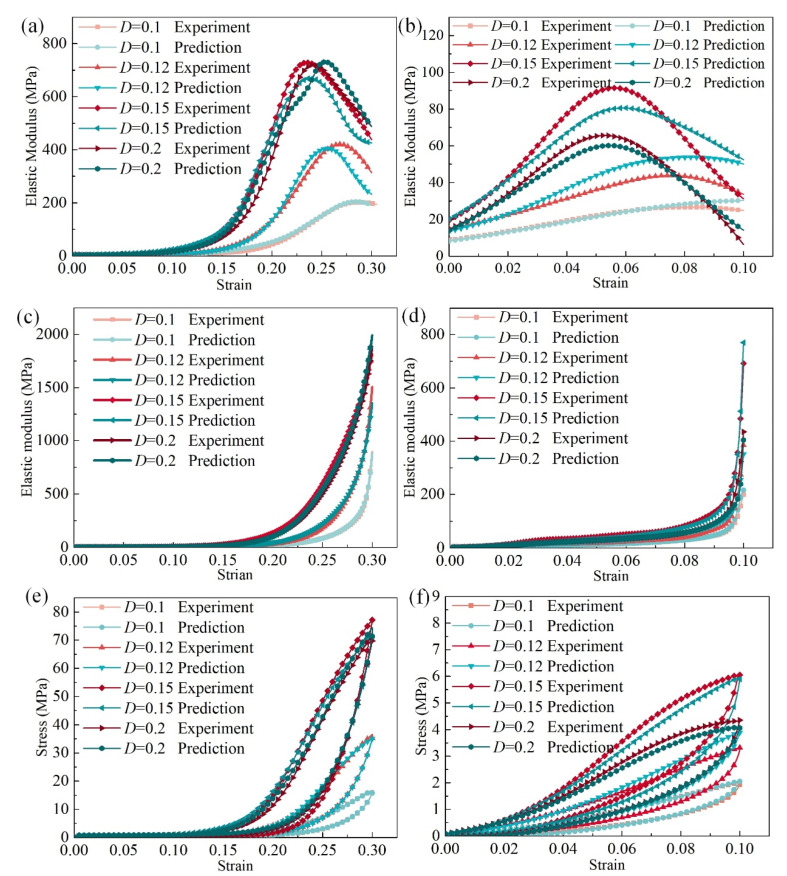
Comparison of the predicted mechanics and experimental results: (**a**) elastic modulus in the loading stage in the *y*–direction, (**b**) elastic modulus in the loading stage in the *x*–direction, (**c**) elastic modulus in the unloading stage in the *y*–direction, (**d**) elastic modulus in the unloading stage in the *x*–direction, (**e**) stress in the *y–*direction, (**f**) stress in the *x*–direction.

The ANN model prediction and experimental results are compared in Figure 9. As shown in Figure 9a, for the elastic modulus in the loading stage and *y*–direction, the ANN model showed great prediction accuracy. The ANN predicted the current elastic modulus in the loading stage, and the *x*–direction (or *z*) showed a similar trend with the experimental results, as observed in Figure 9b. Figure 9c,d shows the unloading elastic modulus in the *y*–direction and *x*–direction (or *z*), respectively.

For the structural FEM analysis, stress is the most important factor, and the purpose of the current elastic modulus is to calculate the nonlinear stress. The predicted stress of the MR cube can be calculated according to the predicted current elastic modulus. Figure 9e compares the stress in the *y*–direction between the ANN prediction and experimental results, and a similar comparison of the stress in the *x*–direction (or *z*) is presented in Figure 9f. The experimental results are consistent with the ANN model prediction, which indicates that the trained ANN model shows great performance and accuracy.

### 3.3. ANN MR Material Model for FEM Analysis

**Figure 10:**
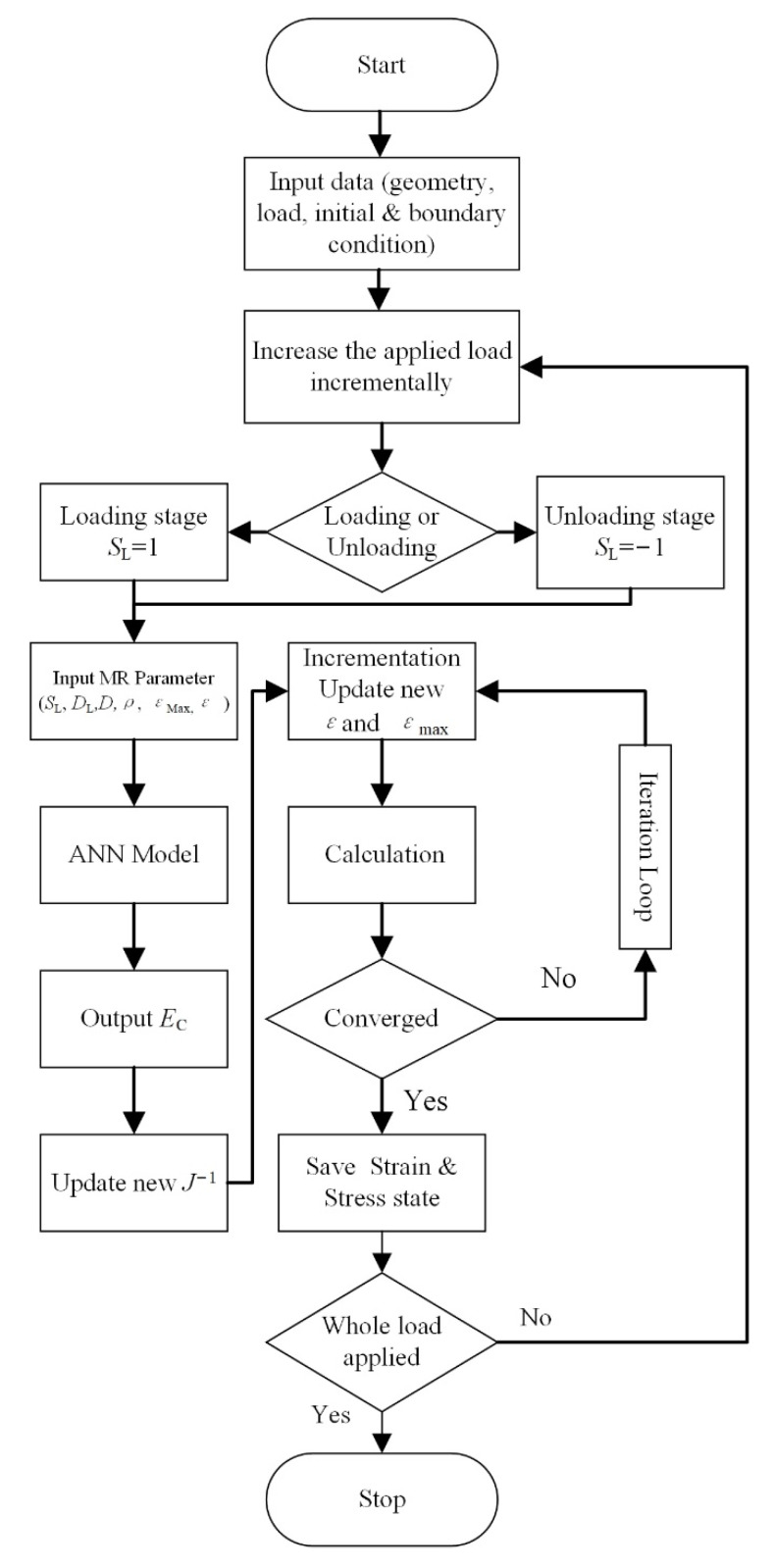
Flow chart of the calculation procedure.

Based on the combination of the ANN model and the FEM, we present a calculation method for the MR components. UMAT and VUMAT interfaces of ABAQUS allow users to define the property of special materials. The elasticity matrix of the MR and ANN models were programmed using the FORTRAN language. The nonlinearity and anisotropic strain–stress property of the MR material were delivered to ABAQUS using the programmed FORTRAN subroutine. Figure 10 shows the flow charts of the calculation procedure.

### 3.4. Static Compresstion of MR Isolator

**Figure 11:**
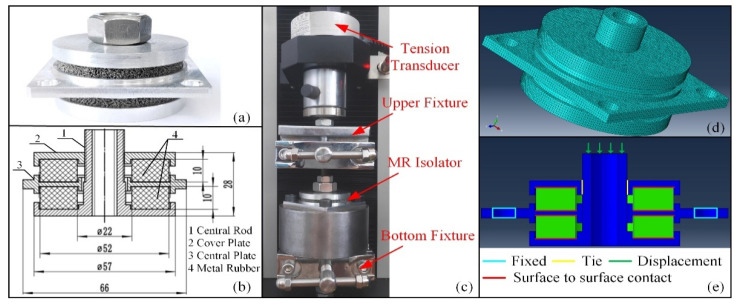
Static experiment and FEM simulation: (**a**) MR isolator, (**b**) assembly diagram of the MR isolator (in millimeter units), (**c**) Static testing equipment, (**d**) FEM model, (**e**) boundary conditions of FEM model.

The static stiffness is the fundamental factor in the capacity design of an MR isolator. In order to demonstrate the availability of the ANN material model of MR, we conducted the static stiffness experiment and FEM analysis of the MR isolator. Figure 11a shows the MR isolator, and Figure 11b is the assembly diagram of the MR isolator. The MR isolator consists of five parts: the central rod, the cover plate, the central plate, and two MR hollow cylinders (*ρ* = 0.22, *D* = 0.1 mm). After the assembly, each MR has a 10% strain preload. The experiments were performed using a universal testing machine (WDW-5Y, Jinan East Testing Machine Co., Ltd., Jinan, China). Figure 11c shows the static testing of the MR isolator. During the experiments, the central plate was fixed by the bottom fixture; the upper fixture clamped the central rod and moved along the axial direction. The force was captured by the tension transducer.

Figure 11d shows the FEM model of the MR isolator. The boundary conditions of the FEM model are as follows: (1) the four holes of the central plate were fixed, (2) the contact between the MR and other parts was defined as a rough surface to surface contact, (3) the connection between the central plate and central rod was simplified as a tie connection, and (4) the displacement load was applied to the end surface of the central rod, as shown in Figure 11e.

**Figure 12:**
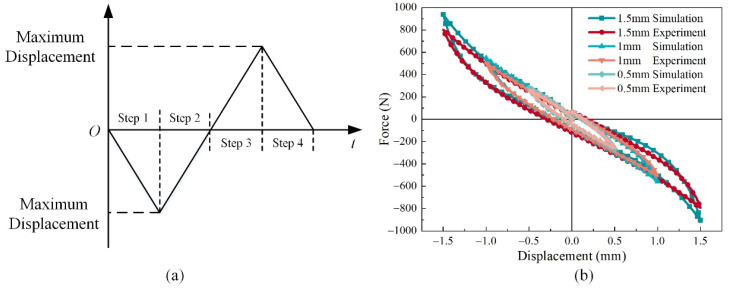
Step and results of static experiments and FEM simulations: (**a**) loading steps schematic, (**b**) results under different maximum displacements.

While the central rod is moving, one of the MRs is loading, and the other one is unloading or static, so the stiffness of the isolator is dependent on the nonlinearity of MR both in the loading and unloading phases. Figure 12a shows the loading steps of the experiments and simulations. We conducted three groups of comparison, and the maximum displacements were 0.5, 1.0, and 1.5 mm, respectively. The two MR hollow cylinders are distributed symmetrically about the middle plane, so the hysteresis loops are almost symmetrically about the original point except for the start point and end point. However, this is the feature of the dry friction damper. The consistency of the results of FEM simulation and experiments testifies that the presented material model correctly expresses the MR’s mechanical property.

### 3.5. Shock Response of MR Isolator

The period of the shock process is very short, and the shock energy will be absorbed by the deformation of the MR; consequently, the acceleration and shock force will be reduced. For shock response, the hysteretic damping *h* caused by dry friction is shown in Equation (12), and the governing equation of dynamic motion for a hysteretic damping system is shown in Equation (13) [57].

(12) $$h=\frac{W_{\mathrm{dis}}}{\pi A^{2}},$$

(13) $$m\ddot{x}+\frac{h}{w}\overset{\cdot }{x}+kx\;=\;F\exp(jwt),$$

where *W*_dis_ is energy dissipated per cycle, *A* is the amplitude of deformation, *m* is the mass, *x* is the displacement of the mass, $\overset{\cdot }{x}$ is velocity, which is the first derivative of displacement with respect to time, $\ddot{x}$ is acceleration, which is the second derivative of displacement with respect to time, *ω* is the angular frequency of the excitation, *k* is the stiffness, *F* is the amplitude of force excitation, *j* is the imaginary unit, and *t* is time.

**Figure 13:**
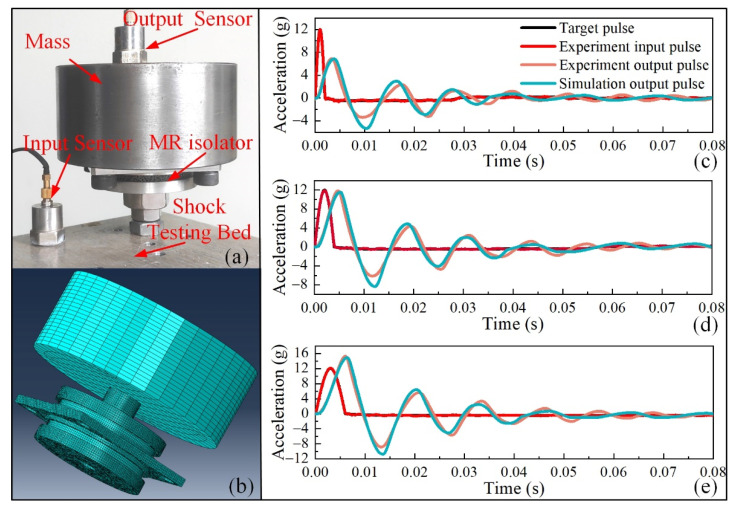
Shock experiment and FEM simulation: (**a**) shock testing system, (**b**) FEM model, (**c**) results of periods of 2 ms, (**d**) results of periods of 4 ms, (**e**) results of periods of 6 ms.

According to Equations (12) and (13), for the MR isolator, the hysteretic damping *h* and stiffness *k* both depend on the mechanics of MR. Therefore, the shock experiments and FEM simulation of the MR isolator were employed to demonstrate the availability of the ANN material model of MR. The experiments were performed using a vibration and shock test system (MPA403/M124A ETS Solution Beijing Ltd., Beijing, China). Figure 13a shows the shock testing system of the MR isolator. The input signal and output responses were collected using acceleration sensors. The mass cylinder (2.5 kg) was the target object that needed to isolate from the shock excitation. The MR isolator was rigidly connected to the shock testing bed by a stud. The mass cylinder was connected to the central plate of the isolator by four bolts.

Figure 13b shows the FEM model for the shock response analysis of the MR isolator. On the basis of the static model, two boundary conditions were modified: (1) the displacement load of the central rod was removed, and the connection between the central rod and the mass cylinder was set as a tie connection, (2) the fixed constraint of the central plate was removed, and the shock excitation was applied to the central plate. We conducted three groups of shock experiments and simulations. The three input shock pulses were all half-sinusoid with 12 g amplitude, but the periods were 2, 4, and 6 ms, respectively. The results of the experiments and FEM simulations are shown in Figure 13c–e. The results indicate that the input impulses were significantly attenuated with time, and after three cycles of vibration, the acceleration was almost reduced to zero. The amplitude of output acceleration increased with the period of shock excitation; for this isolator, the shock was amplified when the period of shock excitation was 6 ms. The response signals of the experiment and simulation fit very well. The consistency between the results of the experiments and FEM simulations indicates the feasibility and accuracy of the MR material model.

The accuracy and efficiency of the presented numerical simulation method rely on the MR materials model. However, the presented ANN model is suitable for the particular type of MR components, which have to match the following three features: (1) the material of the metal wire is 321 stainless steel; (2) the diameter of the micro springs is 10 times that of the metal wire; and (3) there is no significant size effect. Additionally, these limitations can be overcome by an extended ANN model that considers further factors, such as the wire materials, spring diameters, and cycle index.

## 4. Conclusions

We proposed an ANN constitutive model to describe and predict the anisotropic and nonlinear mechanics of MR. First, the anisotropy and nonlinearity of the mechanical properties of the MR were analyzed. The anisotropy stemmed from the different arrangement patterns of micro springs in different directions. The nonlinear feature of MR was reflected in the relationship between the stress and strain, which showed nonlinearity. This feature was affected by the metal wire diameter, relative density, maximum strain, and loading direction. Next, we built, trained, and validated an ANN model to predict the current elastic moduli of the MR precisely. The well-predicted current elastic moduli were transferred to the elasticity matrix to describe the nonlinearity and anisotropy of the MR. In addition, an MR isolator with a relative density of 0.22 and wire diameter of 0.2 mm was simulated and tested by applying a series of half-sinusoid shock waves. The good agreement between the simulated and experimental results confirmed the feasibility and accuracy of the MR ANN model. This model can be readily extended to consider further factors, including the wire materials, spring diameters, and cycle index. This work proposes an efficient and accurate numerical calculation method for the design of MR components.

## Figures and Tables

**Table 1:** Parameters of MR cube samples.

Metal Wire Diameter *D*	*ε*_max_ in *x*–Direction	*ε*_max_ in *y*–Direction	Relative Density *ρ* (Training)	Relative Density *ρ* (Testing)
0.1 mm	0.1, 0.15, 0.2, 0.25, 0.3	0.025, 0.05, 0.075, 0.1	0.23, 0.25, 0.28, 0.3, 0.33, 0.35, 0.37, 0.4, 0.42, 0.45	0.26
0.12 mm	0.1, 0.15, 0.2, 0.25, 0.3	0.025, 0.05, 0.075, 0.1	0.23, 0.25, 0.28, 0.3, 0.33, 0.35, 0.37, 0.4, 0.42, 0.45	0.32
0.15 mm	0.1, 0.15, 0.2, 0.25, 0.3	0.025, 0.05, 0.075, 0.1	0.23, 0.25, 0.28, 0.3, 0.33, 0.35, 0.37, 0.4, 0.42, 0.45	0.39
0.2 mm	0.1, 0.15, 0.2, 0.25, 0.3	0.025, 0.05, 0.075, 0.1	0.23, 0.25, 0.28, 0.3, 0.33, 0.35, 0.37, 0.4, 0.42, 0.45	0.385

**Table 2:** Various structures to optimize the ANN.

No.	Neural Network Structure	Training		Testing	
		MSE	*R* ^2^	MSE	*R* ^2^
1	8 × 4	171	0.9955	446	0.9893
2	16 × 8	17.0	0.9995	376	0.9905
3	24 × 12	2.70	0.9999	292	0.9930
4	32 × 16	0.81	0.9999	439	0.9853
5	8 × 4 × 2	119	0.9962	348	0.9916
6	16 × 8 × 4	11.5	0.9997	366	0.9912
7	24 × 12 × 6	1.06	0.9999	1913	0.9544
8	32 × 16 × 8	0.48	0.9999	2273	0.9458

## Data Availability

The data used in this work are available from the authors upon reasonable request.
